# Expansion of CD4^+^CD25^+ ^helper T cells without regulatory function in smoking and COPD

**DOI:** 10.1186/1465-9921-12-74

**Published:** 2011-06-08

**Authors:** Ester Roos-Engstrand, Jamshid Pourazar, Annelie F Behndig, Anders Bucht, Anders Blomberg

**Affiliations:** 1 Dept. of Public Health and Clinical Medicine, Division of Medicine, Umeå University, Sweden; 2 Swedish Defence Research Agency, Division of CBRN Defence and Security, Umeå, Sweden

**Keywords:** Bronchoalveolar lavage, BAL, CD25^bright^, CD127, FoxP3, lymphocyte subsets

## Abstract

**Background:**

Regulatory T cells have been implicated in the pathogenesis of COPD by the increased expression of CD25 on helper T cells along with enhanced intracellular expression of FoxP3 and low/absent CD127 expression on the cell surface.

**Method:**

Regulatory T cells were investigated in BALF from nine COPD subjects and compared to fourteen smokers with normal lung function and nine never-smokers.

**Results:**

In smokers with normal lung function, the expression of CD25^+^CD4^+ ^was increased, whereas the proportions of FoxP3^+ ^and CD127^+ ^were unchanged compared to never-smokers. Among CD4^+ ^cells expressing high levels of CD25, the proportion of FoxP3^+ ^cells was decreased and the percentage of CD127^+ ^was increased in smokers with normal lung function. CD4^+^CD25^+ ^cells with low/absent CD127 expression were increased in smokers with normal lung function, but not in COPD, when compared to never smokers.

**Conclusion:**

The reduction of FoxP3 expression in BALF from smokers with normal lung function indicates that the increase in CD25 expression is not associated with the expansion of regulatory T cells. Instead, the high CD127 and low FoxP3 expressions implicate a predominantly non-regulatory CD25^+ ^helper T-cell population in smokers and stable COPD. Therefore, we suggest a smoking-induced expansion of predominantly activated airway helper T cells that seem to persist after COPD development.

## Introduction

Chronic obstructive pulmonary disease (COPD) is characterized by progressive airway obstruction and airway inflammation. Tobacco smoking is the main risk factor for COPD. Smoking causes an inflammatory response in all smokers but only 50 percent develop COPD [1].

Increased numbers of neutrophils, macrophages and T lymphocytes have been found in the lungs of COPD patients [2,3]. A relationship has been shown between the number of cytotoxic CD8^+ ^T-cells and a decline in lung function in patients with COPD [4,5] suggesting a role for these cells in the pathogenesis of COPD. The balance between CD4^+ ^helper T cells and CD8^+ ^cytotoxic T-cells is altered in the lungs of COPD patients, which results in a decline in the CD4/CD8 ratio [4]. Both CD4^+ ^and CD8^+ ^cells have been shown to be more activated in both smokers and in subjects with COPD [6]. CD25 is a constitutively expressed activation marker and CD4^+ ^cells with "bright" or "high" expression of CD25^+ ^have been suggested to be regulatory T cells, previously defined as suppressor T cells [7]. Their function is to suppress immune responses by the secretion of soluble inhibitory mediators, such as interleukin 10, or through direct cell-to-cell contact. The role of regulatory T cells in COPD is not well-known, but Smyth et al have reported that long-term cigarette smoking increases airway regulatory T cell numbers, in terms of CD4CD25^bright ^cells [8]. In contrast, two other studies reported decreased levels of regulatory T-cells in subjects with emphysema and COPD compared to healthy controls [9,10].

However, CD25 ^bright ^is not a definite marker of regulatory T cells [11]. Transcription factor fork head box P3, FoxP3, is considered a unique intra-nuclear regulatory T cell marker. A mutation in the FoxP3 gene can cause immune dysfunction polyendocrinopathy enteropathy X-linked syndrome, IPEX, but also other autoimmune conditions such as diabetes, thyreoditis and inflammatory bowel diseases [12]. Recently, absent or low expression of the IL-7α receptor (CD127^dim^) has been reported as another unique marker for regulatory T cells [13]. As CD127 is an extracellular marker, it is more easily analysed compared to FoxP3. Studies have shown that CD127 is down-regulated on all human T cells after activation [14]. In a recent study, we have shown that airway T cells are highly activated in COPD as indicated by increased expression of CD69 and HLA-DR [6]. In addition, CD4^+ ^cells express high levels of CD25 in COPD and smokers, suggesting the presence of regulatory T-cells [6]. It is of importance to verify and evaluate regulatory T cells in COPD in more detail, as these cells may play a role in the pathogenesis of COPD, as suggested by Barceló [10]. The aim of this study was therefore to identify airway regulatory T cells in smokers and individuals with COPD, using flow cytometric analysis of CD127 and FoxP3 and their relation to CD25 expression.

## Materials and methods

### Subjects

Nine patients with COPD (four ex-smokers and five smokers), fourteen smokers with normal lung function (defined as smokers with normal dynamic spirometry, i.e. FEV_1 _and FVC values within 80-120% of predicted value) and nine healthy never-smokers were recruited, (table 1). All COPD subjects and smokers with normal lung function had a smoking history of at least ten pack-years. Current smokers were not allowed to smoke for at least 12 hours prior to bronchoscopy. The subjects were not allowed to have any other medical condition apart from COPD.

**Table 1:** Demographics and spirometry values

	Never-smokers n = 9	Smokers n = 14	COPD Ex-smokers n = 4	COPD Smokers n = 5
**Male:Female**	**5:4**	**7:7**	**4:0**	**0:5**
**Age**	**65 ± 5.2**	**60 ± 6.6**	**67 ± 2.1**	**61 ± 2.4**
**Smoking (pack years)**	**0 (0-0)**	**30 (20-44)**	**50 (44-52)**	**46 (35-65)**
**COPD stage** **(GOLD)^+^**	**NA**	**NA**	**2 and 3**	**2 and 3**
**FEV_1_/FVC %** **Pre bronchodilatation**	**77 (74-83)**	**78 (75-82)**	**62 (60-67)**	**60 (56-67)**
**FEV_1_/FVC %** **Post bronchodilatation**	**NA**	**78 (77-81)**	**64 (62-67)**	**60 (59-66)**
**FEV_1 _% of predicted Pre bronchodilatation**	**101 (89-110)**	**112 (104-120)**	**53 (40-64)**	**53 (47-66)**
**FEV_1 _% of predicted Post bronchodilatation**	**NA**	**114 (108-119)**	**63 (52-67)**	**60 (55-68)**

Data are shown as mean ± standard deviation for age, median and inter quartile range for all others. FEV_1_: Forced expiratory volume in one second; FVC: Forced vital capacity. NA: not applicable. + http://www.goldcopd.org. In the ex-smoking COPD patients, the smoking cessation was more than five years prior to study inclusion.

The COPD patients did not receiv any treatment with inhaled corticosteroids or oral anti-inflammatory drugs during at least four weeks prior to study start and neither regular long-acting β_2_-agonists nor long-acting anti-cholinergic drugs were allowed within two weeks prior to bronchoscopy. Short-acting β_2_-agonists and/or anti-cholinergic drugs were used on demand. All subjects were non-atopic and free from symptomatic airway infection within a six week-period prior to the study. None had a history of chronic bronchitis or frequent infectious exacerbations. All COPD patients had a post bronchodilator FEV_1_/FVC of less than 70% and were not reversible. Informed consent was obtained from all volunteers after verbal and written information and the study was approved by the local Ethics Review Board at Umeå University, Sweden, and performed according to the declaration of Helsinki.

## Methods

### Spirometry

Dynamic spirometry (FVC and FEV_1_) was performed post-bronchodilatation using a Vitalograph spirometer (Vitalograph Ltd., Buckingham, UK), as outlined previously [6].

### Bronchoscopy

Before bronchoscopy atropine was given subcutaneously. Topical anaesthesia of the airways was obtained with lidocaine. All subjects were examined in the supine position using an Olympus BF IT160 video bronchoscope (Olympus, Tokyo, Japan). Bronchoalveolar lavage (BAL) was performed by infusing three aliquots of 60 ml of sterile sodium chloride (NaCl), pH 7.3 at 37°C that were gently sucked back after each infusion and pooled into a container placed in iced water. The recovered fluid was immediately transported to the laboratory for analysis.

### Flow cytometry analysis

BAL -lymphocyte subsets were determined using flow cytometry. BAL cells were centrifuged and diluted to a final concentration of 10^6 ^cells/ml. For each test, 10 μl of antibody solution was added to 200 μl of cell suspension and allowed to bind for 30 minutes at 4°C in darkness. Red blood cells were lysed with 2 ml FACS^™ ^Lysing solution (Becton Dickinson Immunocytometry Systems, San Jose, CA, USA) for 10 minutes at room temperature. The remaining cells were then washed by adding PBS to the tubes and centrifuged at 4°C for 10 minutes, 300 g and repeated once. Cells were thereafter fixed with 500 μl CellFIX^™ ^(Becton Dickinson Immunocytometry Systems, San Jose, CA, USA) before analysis using a FACSCalibur^™ ^(Becton Dickinson) flow cytometer. The lymphocyte population was gated on their physical characteristics in a region according to their characteristic forward scatter (FCS) and side scatter (SSC) profiles, as previously reported [6]. 3,000 total events were collected in CD3^+ ^gate per sample.

**Figure 1:**
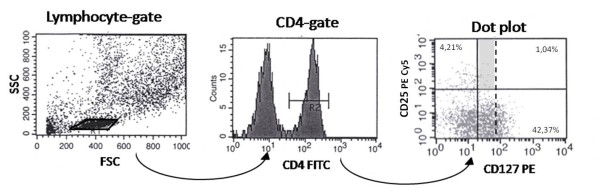
**Flow cytometry analysis of CD127 expression on BAL CD4^+ ^T cells**. Firstly, lymphocytes were gated in FSC and SSC. Secondly, CD4^+ ^cells were gated in the histogram. CD25 and CD127 expression on the gated CD4 cells were analyzed in a dot plot. The grey area indicates CD25^+^CD127^dim ^population.

To identify CD3^+^, CD4^+^, CD25^+ ^and CD127^+ ^cells, the cells were stained with Allophycocyanin (APC) conjugated anti-human CD3, fluorescein isothiocyanate (FITC) conjugated anti-human CD4, phycoerytrin-Cy5 (PE Cy5) conjugated anti-human CD25 and phycoerytrin (PE) conjugated anti-human CD127 in the same test tube (Becton Dickinson, San Jose, CA, USA). The percentage of different cell types was counted out of gated CD3^+ ^lymphocytes and furthermore out of gated CD4^+^. CD25^bright ^cells were quantified as previously described [8,15]. Analyses of CD127^-&dim ^are performed as shown in Figure 1.

Intracellular staining of FoxP3 was conducted according to the recommended procedure obtained from eBioscience (San Diego, CA, USA). Cells were permeabilised with eBioscience FoxP3 Staining Buffer Set at 4°C for 30 minutes. By adding permeabilisation buffer to the tubes, cells were washed and centrifuged at 4°C for 10 minutes, 300 g. This washing procedure was performed twice. 10 μl of antibody solution was added to the cell suspension and allowed to bind for 30 minutes at 4°C in darkness. The cells were washed twice by adding permeabilisation buffer to the tubes and centrifuged at 4°C for 10 minutes, 300 g and 300 μl of PBS was added. To identify CD3^+^, CD4^+ ^and CD25^+ ^cells, the cells were stained with same antibodies as in the extracellular staining. To obtain FoxP3^+ ^cells, phycoerytrin (PE) conjugated anti-human FoxP3 was used in the same test tube. The percentage FoxP3 was determined out of gated CD3^+ ^and CD4^+ ^lymphocytes.

## Statistical analysis

Flow cytometry data were acquired and analysed using CellQuest Software (Becton Dickinson). Differences between three groups were tested using Kruskal-Wallis test and a p-value of less than 0.05 was considered significant. If the Kruskal-Wallis test indicated significance, the Mann-Whitney U-test was used for post-hoc analysis for comparison between two groups, with corrections of p-values according to Bonferroni (a p-value less than 0.017 was considered significant). Whilst the number of COPD patients was small, the ex-smoking COPD group was compared to the smoking COPD group, using Mann-Whitney U-test. Here, a p-value of less than 0.05 was considered significant.

## Results

The BAL recovery in subjects with COPD was (37%; 29-52), (median; inter quartile range) in smokers with normal lung function (53%; 49-61) and in never-smokers (50%; 34-64).

Smokers with normal lung function had increased total leukocyte numbers in BAL compared to never-smokers. Among leukocytes, the macrophage numbers were increased. The number of macrophages was also increased in smokers with normal lung function, compared with COPD patients (table 2).

**Table 2:** Differential cell count of leukocytes in BAL fluid, given in number cells/ml*10^4^

	Never smokers (NS) n = 9	Smokers (S) n = 14	COPD n = 9	p	COPD ex-smokers n = 4	COPD smokers n = 5	p
**Total leukocytes**	21 (13-26)	41 (34-52)	25 (18-37)	P < 0.001 NS vs S	20 (10-26)	29 (22-52)	NS

**Macrophages**	19 (12-23)	37 (31-48)	22 (17-33)	P < 0.001 NS vs S P < 0.014 S vs COPD	19 (9.6-22)	27 (20-42)	P < 0.05 COPD ex-s vs COPD s

**Neutrophils**	0.2 (0.045-0.31)	0.49 (0.23-1.1)	0.19 (0.06-0.93)	NS	0.36 (0.04-1.07)	0.19 (0.07-5.2)	NS

**Lymphocytes**	1.7 (1.1-2.2)	2.3 (1.5-3.9)	1.5 (0.90-2.8)	NS	1.5(0.8-2.2)	1.5 (0.7-4.1)	NS

**Eosinophils**	0.08 (0-0.54)	0.05 (0-0.3)	0.06 (0.01-0.66)	NS	0.015 (0.00-0.52)	0.47 (0.05-0.96)	NS

**Mast cells**	0.06 (0.005-0.12)	0.06 (0.03-0.10)	0.03 (0.005-0.065)	NS	0.005 (0.00-0.04)	0.04 (0.02-0.10)	NS

Data are given as median and IQR.

To examine whether the difference in airway inflammation between COPD patients and smokers with normal lung function was due to smoking habits, the group of COPD patients was divided into current smokers and ex-smokers. This subgroup analysis showed that smoking COPD patients had increased BAL macrophage numbers compared to ex-smokers (table 2).

**Figure 2:**
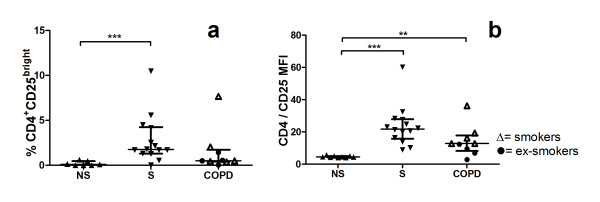
**Flow cytometry analyses of BAL T cells of never-smokers (NS), smokers with normal lung function (S) and COPD**. CD4^+^CD25^bright ^are given as percent of gated CD3 (a). CD25^+ ^cells out of CD4^+ ^cells are given as median fluorescence intensity, MFI (b). Significance levels are noted as ** p < 0.01, *** p < 0.001. Data are given as median and IQR.

**Figure 3:**
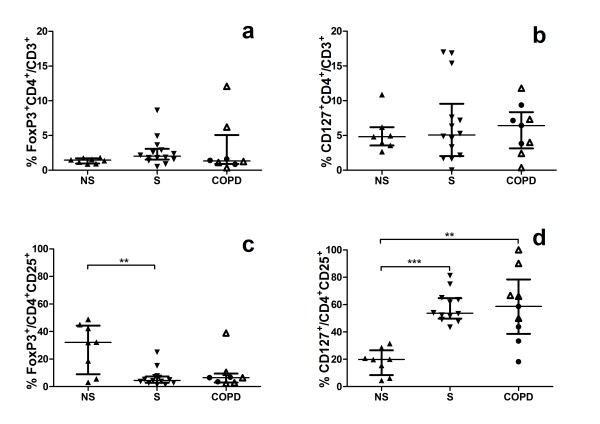
**Flow cytometry analyses of BAL T cells from never-smokers (NS), smokers with normal lung function (S) and COPD subjects**. The proportion of CD4^+ ^T cells expressing FoxP3 (a) or CD127 (b) are given as percent of total T cells (CD3^+^). Percentage of FoxP3^+ ^(c) or CD127^+ ^(d) among CD4^+ ^T cells expressing CD25. The CD127^+ ^population includes the CD127^dim ^cells. Within the COPD group, ● indicates ex-smokers and Δ smokers. Significance levels are noted as ** p < 0.01 and *** p < 0.001. COPD smokers have increased proportions CD127/CD25 among CD4^+ ^cells compared to COPD ex-smokers (p = 0.027) and to never-smokers (p = 0.003). Data are given as median and IQR.

**Figure 4:**
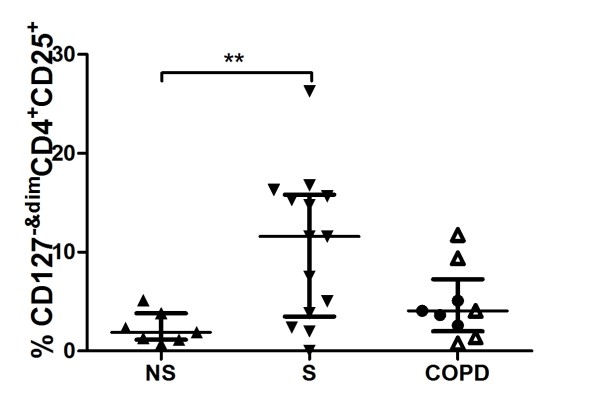
**Flow cytometry analysis of CD127 expression on BAL T cells from never-smokers (NS), smokers with normal lung function (S) and COPD**. The combined CD127^- ^and CD127^dim ^populations are given as percent of gated CD25^+^CD4^+ ^cells. Among the COPD group, ● indicates ex-smokers and Δ smokers. Significance levels are noted as ** p < 0.01 Data are given as median and IQR.

The median fluorescence intensity, MFI, were enhanced in smokers with normal lung function and in COPD, compared to never-smokers (Figure 2). The percentages of CD4^+^CD25^+ ^(data not shown) and CD4^+^CD25^bright ^(Figure 2) cells were enhanced in smokers with normal lung function, compared to never-smokers while the percentage of CD4^+^FoxP3^+ ^and CD4^+^CD127^+ ^cells was unchanged (Figure 3a and 3b). There were no significant difference in CD4^+^CD25^+ ^cells between COPD patients and the other two groups. Among CD4^+ ^T cells expressing CD25, smokers with normal lung function had significantly decreased percentage of FoxP3 compared to never-smokers. CD127 expression on CD4^+ ^T cells expressing CD25 was enhanced in subjects with COPD and smokers with normal lung function, compared to never-smokers (Figure 3). Ex-smoking COPD patients expressed decreased percentage of CD127^+ ^cells in BALF compared to smoking COPD patients (Figure 3d). The expression of CD127^-&dim ^on CD4^+^CD25^+ ^T cells was increased in smokers with normal lung function, compared to non-smokers (Figure 4).

## Discussion

It has been suggested that regulatory T cells are important in the pathogenesis of COPD [8,10]. Recently published data have shown increased CD4^+^CD25^bright ^cells in the airways of subjects with COPD and smokers with normal lung function compared to never-smokers, suggesting the presence of regulatory T cells [6,8]. In contrast, Barceló et al reported decreased percentages of CD4^+^CD25^+ ^in patients with COPD compared to smokers with normal lung function [10] and Lee et al reported similar findings in patients with emphysema [9]. Recently, we found a decreased proportion of CD4^+^CD25^bright ^cells in ex-smoking subjects with COPD compared to smoking COPD subjects [6]. However, despite more than five years after smoking cessation, the proportion of these cells was not normalized, suggesting a smoke-induced upregulation of CD4^+^CD25^bright ^cells. Smoking habits in the other two studies [8,10] were not clearly defined, which makes a full comparison between the studies difficult. CD4^+^CD25^bright ^cells have been suggested to have regulatory features as key immunomodulators. In smokers who maintain normal lung function, it has been implied that the upregulation of regulatory T cells would restrain cigarette smoke-induced inflammatory activation and, thus, the development of COPD [10]. In contrast, in smokers who develop COPD, the T regulatory response is supposed to be inappropriate, which enables an uncontrolled progress of the immunoreaction, involving the activation of T cells into a cytotoxic phenotype. This further supports a potential involvement of the acquired immune response in the pathogenesis of COPD.

To further evaluate the role of regulatory T cells in COPD and to clarify whether CD4^+^CD25^bright ^cells really have regulatory properties, more specific biomarkers are needed. The transcription factor FoxP3 is known to be highly expressed in regulatory T cells, whereas the cell surface marker CD127 is supposed to be low or absent on regulatory T cells [16,17]. Investigations of these markers in COPD are rare and, to our knowledge, this is the first study addressing CD127 expression on BAL cells from smokers and subjects with COPD.

CD127 expressing cells have been studied in allergic asthma, gastric cancer and glioma [13,16,18]. Expression of CD25 and CD127 on CD4^+ ^cells has been suggested to discriminate between regulatory and activated T cells [17]. FoxP3 is strongly expressed in CD25^bright ^cells, whilst CD127 is down-regulated on these cells. CD127 expression is shown to be inversely associated with FoxP3 and suppressive function of human CD4^+ ^regulatory T cells in peripheral blood [14]. In the present study of BAL T cells, a similar pattern was found supporting an inverse association between FoxP3 and CD127 expression also on BAL T cells (Figure 3c, d).

CD25 is of importance in mediating immune tolerance and protection from autoimmune disease [19]. As indicated above, CD25^bright ^expression on CD4^+ ^cells is usually implied as regulatory T cells. The present study shows that an increased percentage of of CD4^+ ^CD25^bright ^cells is associated to current smoking (Figure 2a) and that increased cell surface expression of CD25, expressed as median fluorescence intensity, is associated to both current smoking and COPD (Figure 2b). Even though the number of patients in the present study is rather small, the data are consistent with previously published results [6].

When it comes to the proportion of CD127^+ ^helper T cells among CD3^+ ^cells in BAL fluid, there was no difference between the three groups (Figure 3b). However, in subjects with COPD and smokers with normal lung function, the expression of CD127^+^/CD4^+^CD25^+ ^cells was increased compared to never-smokers (Figure 3d). When COPD subjects were divided into smokers and ex-smokers, we observed that smokers had increased proportions of CD127 on CD4^+^CD25^+ ^cells compared to ex-smokers (Figure 3d). The group of ex-smoking COPD subjects is small, yet the present data imply that tobacco smoking may induce an activation of airway CD4^+ ^cells, in terms of increased CD127 expression and that the CD127 expression appears to decline after smoking cessation. Despite more than five years since smoking cessation, the expression of CD127 among the CD25 helper T cells tended to be higher in COPD patients compared to never-smokers, indicating a prolonged immune activation.

No differences were found between the groups in helper T cells expressing FoxP3^+ ^(Figure 3a). Among CD25 expressing helper T cells, the percentage of FoxP3^+ ^was decreased in smokers compared to never-smokers. The data suggest that a large proportion of CD4^+^CD25^+ ^cells in smokers do not express FoxP3 and, thus, have not a regulatory T cell function. Compared to smokers and non-smokers, a decrease in the expression of FoxP3 has been found in the smaller airways in COPD, whereas FoxP3 expression was increased in large airways in both smokers and subjects with COPD [20]. Another study reported increased regulatory T cell numbers in lymphoid follicles and bronchial tissue in subjects with moderate COPD [21]. Within the lung tissue, regulatory T cells expressing FoxP3 seem to be more abundant in larger airways compared to smaller airways. However, the role for FoxP3 in regulating the immune defence in different regions of the lungs in smoking and COPD needs to be further elucidated.

CD25^+^CD127^dim ^cells are suggested to have immunoregulatory properties, whilst CD25^+^CD127^bright ^have not [17]. Here, the proportion of CD4^+^CD25^+ ^with low or absent expression of CD127 was increased in smokers with normal lung function compared to non-smokers. However, from our data (Figure 4), it appears that the smokers might be divided into two subpopulations, one with increased CD127^-&dim ^on CD4^+^CD25^+ ^cells and one subpopulation with unchanged CD127^-&dim ^expression, suggesting an increased presence of regulatory T cells in some "healthy" smokers. Based on the present data, we hypothesise that, within the group of smokers with normal lung function, there may be subjects with insufficient expansion of regulatory T cells, who will be at risk for developing COPD [1]. If this was the case, it would be possible to distinguish between smoking subjects with different susceptibility to develop COPD. This issue needs to be addressed in future prospective studies. It cannot be excluded that T-lymphocytes isolated from peripheral blood or other lung compartments, such as bronchial mucosa or peripheral lung tissue, may show different phenotypic characteristics compared with BAL-cells. It has been suggested that lung lymphoid tissue contains more T regulatory cells in COPD compared to smokers and healthy subjects [21].

The COPD subjects included in this study were clinically stable, i.e. with no history of recurrent infectious exacerbations and in no need of regular medications, apart from short acting bronchodilators on demand. Also, the ex-smoking COPD subjects stopped smoking more than five years prior to study inclusion, whereas all smokers with normal lung function were current smokers, with at least a smoking history of ten pack years.

The differential cell count confirms previously published data [6]. Macrophages were increased in smokers with normal lung function and in smoking patients with COPD. This is not surprising as macrophages play a key role in the inflammatory response to noxious particles and gases, such as tobacco smoke exposure. The lack of increase in neutrophils in the COPD subjects further implies that these subjects were without any history of bronchitis or frequent infectious exacerbations. There was no difference in lymphocyte numbers between the three groups; the difference was within the lymphocyte population, mainly related to the T lymphocyte subtypes.

In conclusion, we demonstrate that smoking subjects with COPD have increased proportions of CD127^+ ^helper T cells in the airways. Smoking cessation may reduce the proportion of these cells but this has to be confirmed in longitudinal studies. These data therefore indicate the expansion of a T cell population without a regulatory function, which may contribute to the persistent cytotoxic T cells responses previously reported in COPD. However, a fraction of smokers without clinical signs of COPD had an increased population of helper T cells with low or absent CD127 expression, suggesting the presence of regulatory T cells that potentially can modulate the smoke-induced immune responses. Whether such a T cell population would play a role in the protection of COPD development in smokers remains to be elucidated.

## Competing interests

The authors declare that they have no competing interests.

## Authors' contributions

ERE was responsible for preparation and analysis of BAL-samples, statistical analyses, evaluation of data and manuscript preparation. JP contributed with scientific know-how of FACS analyses. AFB took part in subject recruitment, bronchoscopies and manuscript preparation. ABu contributed with scientific expertise and manuscript preparation. ABl was responsible for study design, subject recruitment, bronchoscopies and manuscript preparation.

All authors read and approved the final manuscript.
